# Supplementary material for: MetaRibo-Seq measures translation in microbiomes
Source: Nat Commun. 2020 Jun 29;11:3268. doi: 10.1038/s41467-020-17081-z (PMC7324362; doi:10.1038/s41467-020-17081-z)
Supplement: Supplementary file 10 — Supplementary Data 7 [file 41467_2020_17081_MOESM10_ESM.zip › File2/Confidence_VeryHigh_Taxonomy/59827_out.krona.html]

Javascript must be enabled to view this page.

members
magnitude
magnitudeUnassigned
count
unassigned
taxon
rank

59827\_out

4

4
2
superkingdom

phylum
4
1239

class
3
186801

3
186802
order

186806
2
family

1730
2
genus


SRS013476\_contig\_number\_contig-100\_13247.13247SRS098644\_contig\_number\_14325
species
165185
2

1
541000
family

1924105
1
genus

1
1776382
species

SRS013965\_contig\_number\_contig-100\_22286.22287

526524
1
class

1
526525
order

family
1
128827

genus
1472649
1

1
1034346
species

SRS023715\_contig\_number\_20328
